# Reduced norovirus epidemic follows increased sales of hand hygiene products in Japan, 2020–2021

**DOI:** 10.1265/ehpm.22-00155

**Published:** 2023-03-03

**Authors:** Shinako Inaida, Atsushi Mizukoshi, Kenich Azuma, Jiro Okumura

**Affiliations:** Department of Environmental Medicine and Behavioral Science, Faculty of Medicine, Kindai University

**Keywords:** Gastroenteritis, Surveillance, Hand sanitizer, Pandemic

## Abstract

During the recent emergence of COVID-19, an increased practice of hand hygiene coincided with the reduced incidence of the norovirus epidemic in Japan, which is similar to experience with the pandemic flu in 2009. We investigated the relationship between the sales of hand hygiene products, including liquid hand soap and alcohol-based hand sanitizer, and the trend of norovirus epidemic. We used national gastroenteritis surveillance data across Japan in 2020 and 2021 and compared the base statistics of incidence of these two years with the average of the previous 10 years (2010–2019). We calculated the correlations (Spearman’s Rho) between monthly sales of hand hygiene products and monthly norovirus cases and fitted them to a regression model. In 2020, there was no epidemic, and the incidence peak was the lowest in recent norovirus epidemics. In 2021, the incidence peak was delayed for five weeks to the usual epidemic seasons. Correlation coefficients between monthly sales of liquid hand soap and skin antiseptics and norovirus incidence showed a significantly negative correlation (Spearman’s Rho = −0.88 and *p* = 0.002 for liquid hand soap; Spearman’s Rho = −0.81 and *p* = 0.007 for skin antiseptics). Exponential regression models were fitted between the sales of each hand hygiene product and norovirus cases, respectively. The results suggest hand hygiene using these products is a potentially useful prevention method against norovirus epidemics. Effective ways of hand hygiene for increasing the prevention of norovirus should therefore be studied.

**Supplementary information:**

The online version contains supplementary material available at https://doi.org/10.1265/ehpm.22-00155.

## Introduction

Norovirus is a major cause of viral gastroenteritis epidemics that occur between the autumn and winter seasons in Japan [1–3]. Recurrent norovirus epidemic patterns have shown an onset of the epidemic from the beginning of September and a peak in December [1, 3]. Children and elderly people are especially at risk of severe symptoms, such as dehydration, and death [4, 5]. Among the virus variants, infections to GII.4 present more severity than other variants [4, 6, 7]. The predominant norovirus variant in Japan has been GII.4 since the 2006–2007 season, except in the 2010–2011 season when it was GII.3 and in the 2016–2017 and 2020–2021 seasons when it was GII.2 [1, 8, 9]. The transmission of norovirus is mainly through fecal/oral-to-mouth in person-to-person contact; thus, hand hygiene is thought to be a preventive method [10–12]. However, because presently norovirus cannot be cultured in vitro, effective preventative methods need further investigation, particularly through epidemiology.

Meanwhile, during the current COVID-19 period, a decrease in epidemics of several common infectious diseases was observed and thought to be related to increased hygiene [13]. During the emergence of COVID-19, intensive hand hygiene as an individual prevention method of infection, in combination with mask wearing, was widely conducted in Japan [14]. Previously, in 2009, during the emergence of the pandemic influenza, there was a similar event that increased the practice of hand hygiene. That period was associated with a delay of the norovirus epidemic for the first time in the history of gastroenteritis surveillance in Japan [1]. There was a significant negative correlation between the monthly sales of hand hygiene products, namely, hand soap and skin antiseptics (mainly consisting of alcohol-based hand sanitizer), and the monthly number of norovirus cases; when the pandemic peak of swine influenza was over, the sale of these hand hygiene products slowed down, and the norovirus epidemic season started but with a 3-month delay, suggesting hand hygiene suppressed the epidemic of norovirus [1].

In this study, we focused on the effect of hand hygiene for the prevention of norovirus. We investigated the relationship between the sales of hand hygiene products, as a surrogate index of hand hygiene, and the trend of the norovirus epidemic by using the national gastroenteritis surveillance data across Japan in 2020 and 2021.

## Case report

### Methods

We investigated if increased hand hygiene during the COVID-19 period affected the norovirus epidemic by comparing yearly and monthly sales of hand hygiene products, as a surrogate index of hand hygiene, and the yearly and monthly trends of norovirus incidence.

Norovirus incidence data in Japan have been collected through the gastroenteritis surveillance involving over 3000 sentinel clinics (paediatric clinics) that report the weekly number of gastroenteritis cases to the National Institute of Infectious Diseases (NIID) through the prefectural government [15]. The epidemic level has been monitored using weekly gastroenteritis cases, which define the incident cases per sentinel site (hereinafter, incidence). In other words, the epidemic level does not represent the raw number of cases but the cases per sentinel site. RT-PCR testing of epidemic virus variants was conducted for about 10% of all cases, and the results are reported on the NIID website [16]. Thus, although the surveillance includes other causes of gastroenteritis, including bacteria and other viruses such as rotavirus, through the result of RT-PCR testing the norovirus epidemic season has been detected to occur between September and January and peak in December [1, 3].

Using those data, we compared the base statistics of the incidence: the peak week, accumulated number of incidence, and start week of the norovirus epidemic between the two seasons and the average of the previous 10 years before the epidemic of COVID-19 (between the 2010–2011 and the 2019–2020 seasons). The threshold of the start of the norovirus epidemic was set when the weekly number of incidence (average number of incident cases per sentinel site) was over 4 after September [1, 3, 15]. Correlations (Spearman’s Rho) between the monthly sales of hand soap and skin antiseptics (mainly consisting of alcohol-based hand sanitizer) from the Statistical Survey on Trends in Pharmaceutical Production reported from the Ministry of Health, Labour, and Welfare, and norovirus incidence were calculated, and an exponential regression model was fitted to these variables. Yearly sales of hand soap and skin antiseptics were tracked, and these indices were compared with the preceding three years, 2017–2019.

Statistical analysis was performed using SPSS v21 (IBM Corporation, New York, USA). The level of significance was set at 5%.

### Results

**Fig. 1:**
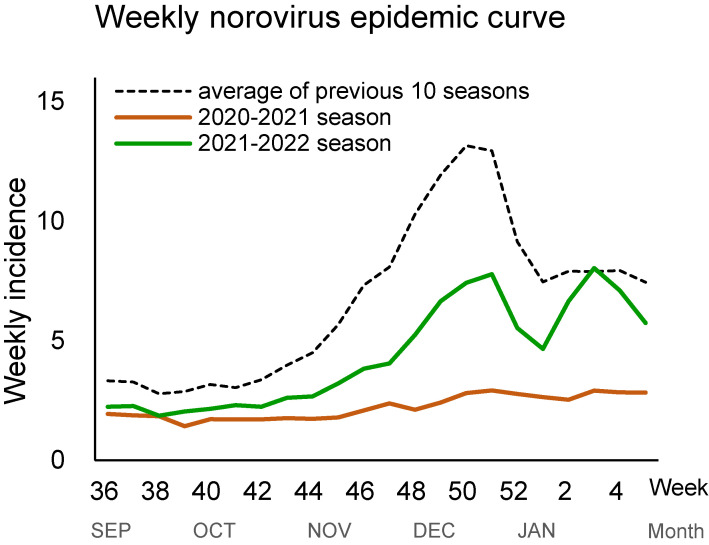
Norovirus epidemic weekly incidence between the 2010–2011 and 2021–2022 seasons. The weekly average incidence (number of cases per sentinel site by week) is shown as grey dashed (2010–2019), solid red (2020–2021 season), and solid blue (2021–2022 season) lines.

An analysis of norovirus data 10 epidemic seasons before the epidemic of COVID-19, between the 2010–2011 and 2019–2020 seasons, showed the start of the epidemic in the 44th epidemiological week and the incidence peak at the 50th week, with a maximum of 13.16 cases per sentinel site (Fig. 1, Table 1).

**Table 1:** Base statistics of norovirus incidence between 2010 and 2022

	**Average of recent ** **10 seasons^c^**	**2020–2021 season**	**2021–2022 season**
Accumulated number of incidence^a^	147.46	48.74	96.29
Maximum (peak)	13.16	2.92	8.04
Minimum	2.78	1.43	1.87
Average [95% CI]*	6.70 [5.21–8.20]	2.22 [2.00–2.43]	4.38 [3.42–5.33]
SD	3.37	0.49	2.16
*Threshold week^b^*	44^th^ week	N/A	47^th^ week
*Peak week*	50^th^ week	51^th^ week	3^rd^ week

*Confidence intervals

^a^Total incidence between September and January

^b^Epidemiological week of outnumbering 4 (the number of cases per sentinel site)

^c^Between the 2010–2011 and the 2019–2020 seasons

In the 2020–2021 season, there was no epidemic, and the number of incidence remained lower than 4 throughout. The incidence peak was at the 51st week in 2020, with a maximum of 2.91 cases per sentinel site, which was the lowest in recent norovirus epidemics in Japan (Table 1). In the 2021–2022 season, the start of the epidemic was delayed until the 47th epidemiological week in 2021, and the incidence peak was delayed until the 3rd week in 2022, with a maximum of 8.04 cases per sentinel site (Table 1). Also, there was a major decrease in incidence during the interim epidemic period, which was around the end of 2021 (Fig. 1).

**Fig. 2:**
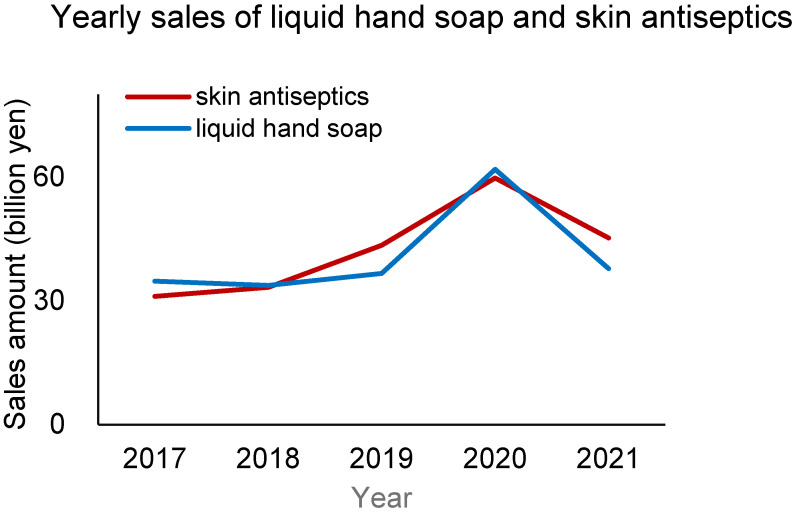
Yearly sales of liquid hand soap and skin antiseptics. The data were reviewed from the open-source of the government [in Japanese]. https://www.meti.go.jp/statistics/tyo/seidou/result/ichiran/08_seidou.html#menu2 (liquid hand soap) https://www.mhlw.go.jp/toukei/list/105-1.html (skin antiseptics) (The sales amount of skin antiseptics were reviewed from the output data.)

Coincidently, yearly sales of liquid hand soap and skin antiseptics increased in 2020 but then decreased in 2021 (Fig. 2).

**Fig. 3:**
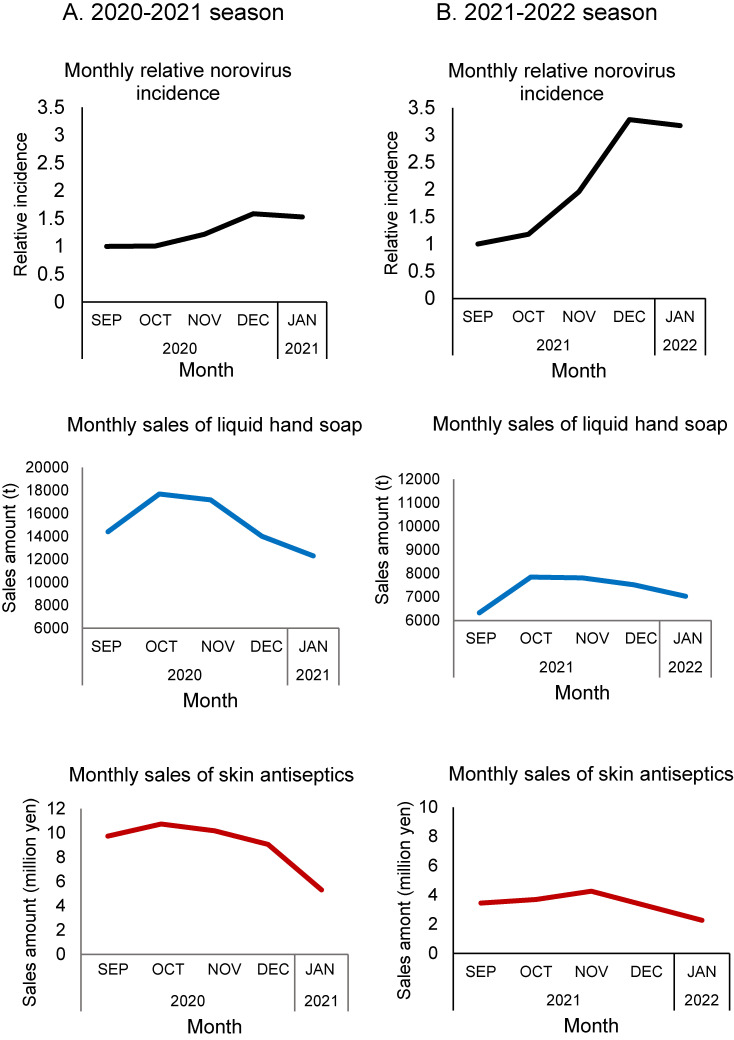
Monthly relative incidence of norovirus and sales of hand hygiene products. Relative monthly incidence against the reference incidence in September and total sales in yen of liquid hand soap and skin antiseptics in A) 2020–2021 season and B) 2021–2022 season. The data were reviewed from the open-source of the government [in Japanese]. https://www.meti.go.jp/statistics/tyo/seidou/result/ichiran/08_seidou.html#menu2 (liquid hand soap) https://www.mhlw.go.jp/toukei/list/105-1.html (skin antiseptics)

**Fig. 4:**
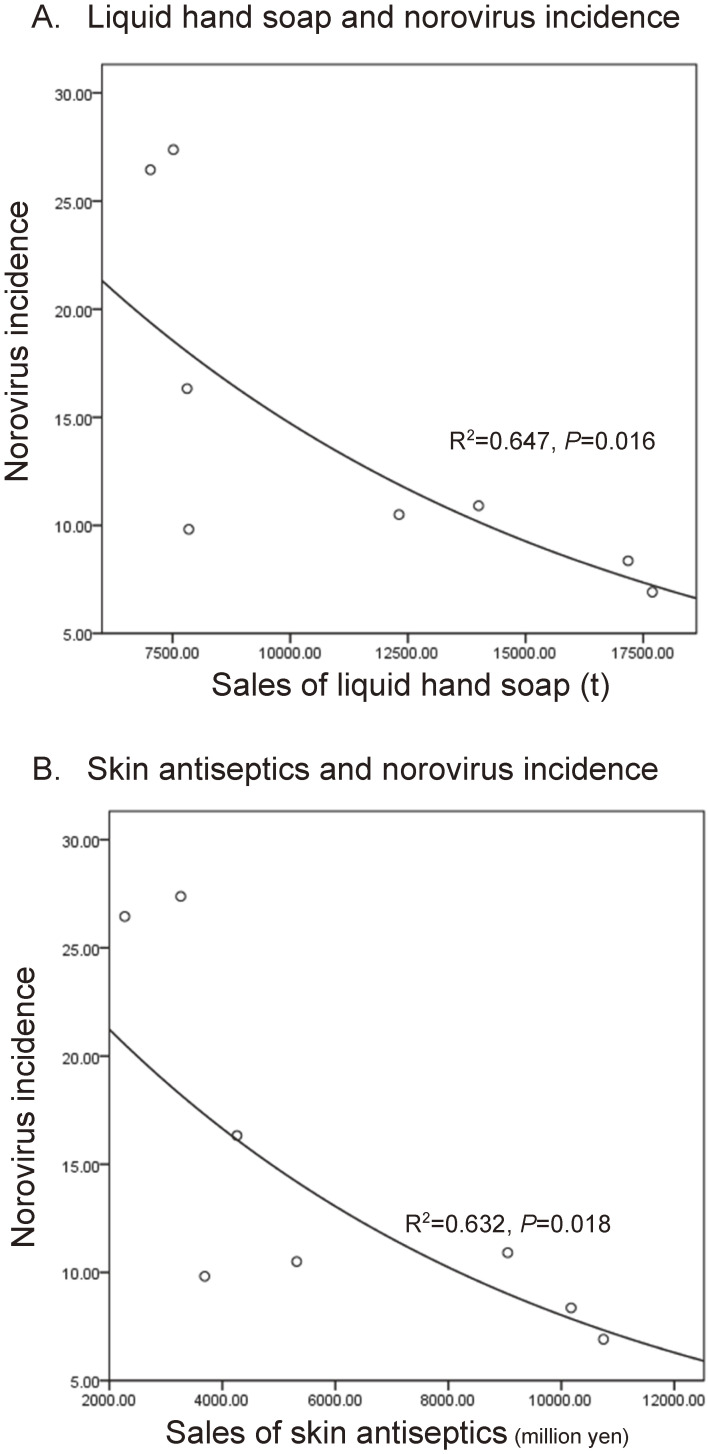
Exponential regression for the incidence of norovirus and sales of hand hygiene products. Sales and incidence data were fitted to exponential regression models for A) liquid hand soap and B) skin antiseptics for the epidemic period of COVID-19 (2020–2021 and 2021–2022 seasons combined). Because the epidemic starts in September, the data in September were excluded in the regression model.

Monthly sales of both liquid hand soap and skin antiseptics increased in the 2020–2021 season but decreased in the 2021–2022 season (Fig. 3). Through the 2020–2021 and the 2021–2022 seasons, the monthly incidence of norovirus was negatively correlated with the sales of hand hygiene products (Spearman’s Rho = −0.88 and *p* = 0.002 for liquid hand soap; Spearman’s Rho = −0.81 and *p* = 0.007 for skin antiseptics; one sided, excluding the data in September because the epidemic starts in September). Exponential regression models showed a negative correlation between the sales of hand hygiene products and norovirus incidence (Fig. 4).

Among the epidemic seasons between the 2017–2018 and the 2021–2022 seasons (five epidemic seasons including three epidemic seasons before the COVID-19), the monthly norovirus incidence sharply decreased after December except for the COVID-19 period, when it plateaued (Fig. 3, Supplementary Figure S1). Monthly sales of liquid hand soap in the 2021–2022 season were similar to the 2017–2020 seasons, but sales in the 2020–2021 season were higher (Supplementary Figure S2). In contrast, monthly sales of skin antiseptics were higher in both the 2020–2021 and 2021–2022 seasons (Supplementary Figure S3). In seasons before the COVID-19 period, there was no relationship between the norovirus incidence and the sales of these hand hygiene products (Supplementary Figure S4).

### Discussion

We found that a decrease of norovirus cases coincided with an increase of hand hygiene in Japan. During the COVID-19 epidemic, there were more than several infectious diseases that showed lower incidence [13]. This observation was attributed to the overall increase in individual hygiene, including not just hand hygiene, but also the cleaning of contact surfaces in public spaces, the wearing of masks, and social distancing. Because one transmission route of norovirus is thought to be finger contact from food handlers [17, 18], the avoidance of eating in restaurants may have reduced the transmission. Likewise, during the COVID-19 pandemic, people were encouraged to avoid social gatherings [19]. Thus, the rate of social contact was lower, which would have decreased the transmission of norovirus. A major decrease in incidence around the end of 2021 suggested that the potential decrease in social contacts during the holiday season that decreased norovirus infections. These backgrounds are likely confounders on the effects of hand hygiene on the decreased incidence of norovirus.

Monthly sales of liquid hand soap in the 2021–2022 season were similar to the 2017–2020 seasons, but sales of liquid hand soap in the 2020–2021 season were higher than the previous seasons, and monthly sales of skin antiseptics were higher in both the 2020–2021 and the 2021–2022 seasons than the previous seasons. Thus, the 2021–2022 season, which is the second epidemic season of norovirus in the COVID-19 period, can likely be a season to examine the effect of skin antiseptics. In addition, during the three epidemic seasons before the COVID-19, when the amount of sales of hand hygiene products had been relatively low, there was no relationship between the sales of hand hygiene products and the incidence of norovirus. These results suggested the increased rate of hand hygiene, that reached such increase in the sales of hand hygiene products, and that could prevent the norovirus epidemics. Furthermore, because of the suppression of norovirus epidemic in the 2021–2022 season, the hand hygiene with skin antiseptics alone could potentially have a preventive effect on norovirus epidemics. Until now, it has not been possible to culture norovirus; thus, we cannot test which hand hygiene, hand washing with soap or alcohol-based hand sanitizer, was more effective for the prevention of norovirus infection. Effective ways of hand hygiene for increasing the prevention of norovirus should therefore be studied. The sale of hand hygiene products fell from the 2020–2021 season to 2021–2022 season. This decrease is attributed to people adapting to the COVID-19 epidemic after one year and thus giving less attention to hand hygiene. This change in hand hygiene may partially explain the norovirus incidence increase from the 2020–2021 season to 2021–2022 season, but more social contact may also be a factor. We used the sales of hand hygiene products as a surrogate index of hand hygiene practice, but data on actual hygiene activity using these products are not available. Nor are there data on the actual amount of hand hygiene products used. Further study is needed to identify best hygienic practices for the prevention of the norovirus epidemic.

## Supplementary information

**Additional file 1:** **Supplementary Figure S1.** Monthly norovirus incidence between 2017–2018 and 2021–2022 seasons. **Supplementary Figure S2.** Monthly sales of liquid hand soap between 2017–2018 and 2021–2022 seasons. **Supplementary Figure S3.** Monthly sales of skin antiseptics between 2017–2018 and 2021–2022 seasons. **Supplementary Figure S4.** Exponential regression for the incidence of norovirus and sales of hand hygiene products before the COVID-19 epidemic (between 2017–2018 and 2019–2020 seasons combined).

## Declaration

### Availability of data and material

All data used in this study were open source and available online.
