# Supplementary material for: Reduced norovirus epidemic follows increased sales of hand hygiene products in Japan, 2020–2021
Source: Environ Health Prev Med. 2023 Mar 3;28:18. doi: 10.1265/ehpm.22-00155 (PMC10025861; doi:10.1265/ehpm.22-00155)
Supplement: Supplementary file 1 — Additional file 1: Supplementary Figure S1. Monthly norovirus incidence between 2017–2018 and 2021–2022 seasons. Supplementary Figure S2. Monthly sales of liquid hand soap between 2017–2018 and 2021–2022 seasons. Supplementary Figure S3. Monthly sales of skin antiseptics between 2017–2018 and 2021–2022 seasons. Supplementary Figure S4. Exponential regression for the incidence of norovirus and sales of hand hygiene products before the COVID-19 epidemic (between 2017–2018 and 2019–2020 seasons combined). [file ehpm-28-018-s001.docx]

Supplementary Figure S1. Monthly norovirus incidence between 2017-2018 and 2021-2022 seasons.

Season

Supplementary Figure S2. Monthly sales of liquid hand soap between 2017-2018 and 2021-2022 seasons.

Season

Supplementary Figure S3. Monthly sales of skin antiseptics between 2017-2018 and 2021-2022 seasons.

Season

Supplementary Figure S4. Exponential regression for the incidence of norovirus and sales of hand hygiene products before the COVID-19 epidemic (between 2017-2018 and 2019-2020 seasons combined).

1. Liquid hand soap and norovirus incidence


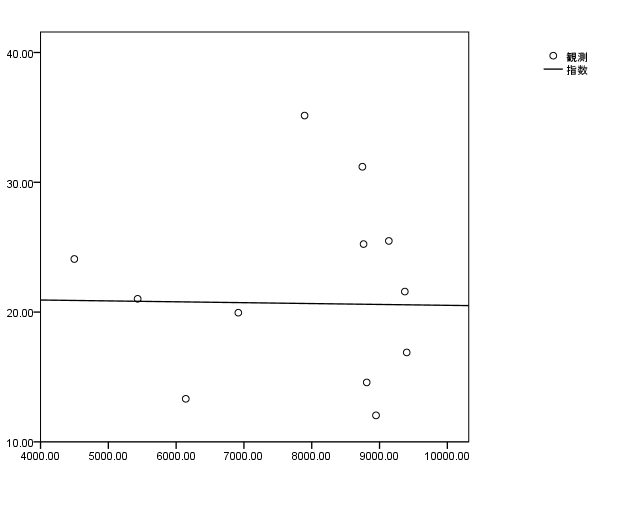


R^2^=0.0001, *P*=0.96

Norovirus incidence

Sales of liquid hand soap (t)

B. Skin antiseptics and norovirus incidence


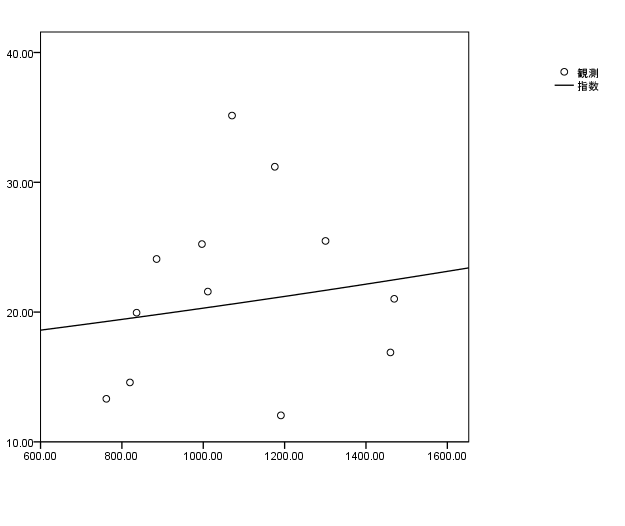


Sales of skin antiseptics (million yen)

Norovirus incidence

R^2^=0.03, *P*=0.62
